# Estimating the potential health and economic impacts of new tuberculosis vaccines under varying delivery strategies in Delhi and Gujarat, India: a modelling study

**DOI:** 10.1016/j.lansea.2024.100424

**Published:** 2024-05-16

**Authors:** Rebecca A. Clark, Allison Portnoy, Chathika K. Weerasuriya, Tom Sumner, Roel Bakker, Rebecca C. Harris, Kirankumar Rade, Sanjay Kumar Mattoo, Dheeraj Tumu, Nicolas A. Menzies, Richard G. White

**Affiliations:** a TB Modelling Group and TB Centre, LSHTM, UK; b Centre for the Mathematical Modelling of Infectious Diseases, LSHTM, UK; c Department of Infectious Disease Epidemiology, LSHTM, UK; d Vaccine Centre, LSHTM, UK; e Department of Global Health, Boston University School of Public Health, USA; f Center for Health Decision Science, Harvard T.H. Chan School of Public Health, USA; g KNCV Tuberculosis Foundation, Netherlands; h Sanofi Pasteur, Singapore; i World Health Organization, India; j International Technical Consultant, The StopTB Partnership, New Delhi, India; k Central TB Division, National Tuberculosis Elimination Program, MoHFW Govt of India, New Delhi, India; l Department of Global Health and Population, Harvard T.H. Chan School of Public Health, USA

**Keywords:** Tuberculosis, Vaccines, Mathematical modelling, Health economics, Cost-effectiveness, India, Subnational

## Abstract

**Background:**

India has the largest tuberculosis burden, but the all-age prevalence in 2021 ranged from 747/100,000 in Delhi to 137/100,000 in Gujarat. No modelling studies have compared the potential impact of new tuberculosis vaccines in regions with differing disease and infection prevalence.

**Methods:**

We used modelling to simulate hypothetical scenarios of introducing M72/AS01_E_ (with 50% efficacy to prevent disease) and BCG-revaccination (with 45% efficacy to prevent infection) in Delhi and Gujarat.

**Findings:**

The hypothetical M72/AS01_E_ scenario could avert 16.0% of cases and 14.4% of deaths in Delhi, and 8.5% of cases and 7.6% of deaths in Gujarat between 2025 and 2050. The hypothetical BCG-revaccination scenario could avert 8.8% of cases and 8.3% of deaths in Delhi, and 5.1% of cases and 4.8% of deaths in Gujarat between 2025 and 2050.

**Interpretation:**

Additional trials for both vaccines are underway, which will provide further evidence on the vaccine efficacy and narrow the range of uncertainty on the estimates.

**Funding:**

10.13039/100000865Bill & Melinda Gates Foundation (INV-001754).

**Research in context**

**Evidence before this study**

The National Tuberculosis (TB) Prevalence Survey in India conducted between 2019 and 2021 estimated an overall disease prevalence of 312 per 100,000, but also indicated that this burden varied widely across the country. The National Capital Territory of Delhi was estimated to have the highest prevalence of disease for all ages (747 per 100,000), while Gujarat was estimated to have the lowest (137 per 100,000). New tuberculosis vaccines are likely to play a key role in tuberculosis elimination and in particular, promising results were reported from Phase IIb trials of M72/AS01_E_ and BCG-revaccination. It is unknown how the impact of delivery of specific vaccine candidates in India may vary depending on differences in subnational demography or burden of disease.

We searched PubMed with no date or language restrictions for all studies modelling the impact of specific tuberculosis vaccine candidates in Delhi or Gujarat using the search terms ((“tuberculosis”) OR (“Mtb”)) AND ((“M72/AS01”) OR (“BCG”)) AND (“India”) AND ((“Delhi”) OR (“Gujarat”) OR (“subnational”)). Studies have estimated that introducing new tuberculosis vaccines could have a positive impact on the epidemic and be cost-effective in India overall, but there were no studies that estimated the impact of new tuberculosis vaccines for regions within India with differing demography and disease burden.

**Added value of this study**

We used mathematical modelling to simulate hypothetical scenarios and investigate how the health impact and cost-effectiveness of M72/AS01_E_ and BCG-revaccination could vary between high- and low-tuberculosis burden areas in India—represented by Delhi and Gujarat—under varying delivery strategies and assumptions on vaccine characteristics aligned with results from the Phase IIb trials, particularly vaccine efficacy. We fit each model to the regional disease prevalence estimated by the National TB Prevalence survey, and, assuming disease prevalence correlated with infection prevalence, modelled a higher infection prevalence in Delhi than in Gujarat.

This was the first modelling study to estimate and compare the impact of introducing novel tuberculosis vaccines for two subnational regions within India which represented high and low burdens of disease. M72/AS01_E_ scenarios were estimated to avert a larger number of cases and deaths in both Delhi and Gujarat compared to BCG-revaccination due to the assumed vaccine characteristics, and more cases and deaths were averted in Delhi compared to Gujarat for both vaccines due to the higher burden of disease. We showed how vaccine impact was closely tied to infection prevalence, given assumptions surrounding vaccines that will be effective only if people are uninfected or if they are infected. As BCG-revaccination was assumed to only be efficacious in those that are uninfected, we estimated a higher relative impact of the vaccine in Gujarat with the lower modelled infection prevalence. Given the assumed vaccine and delivery characteristics, M72/AS01_E_ and BCG-revaccination scenarios were likely to be cost-effective (or even cost-saving) in Delhi. BCG-revaccination scenarios were also estimated to be cost-effective in Gujarat, but M72/AS01_E_ scenarios were likely to be cost-effective only if, given the lower prevalence of infection, we assumed that vaccine efficacy was not restricted to current infection at the time of vaccination.

**Implications of all the available evidence**

Evidence from this study, combined with previous evidence for India overall, continued to show that, given the assumptions on vaccine characteristics, such as the vaccine efficacy, and delivery strategies, new tuberculosis vaccines could be impactful and cost-effective when introduced. Moving forward, age-specific regional estimates of *Mtb* infection prevalence are needed to better inform vaccine impact estimation for vaccines that may only be effective if individuals are either uninfected or infected. Determining whether M72/AS01_E_ is able to prevent both infection and disease, and if it is efficacious in those that are uninfected at the time of vaccination is an additional area for continued research to reduce unknowns.

## Introduction

India has the highest global burden of tuberculosis, but this burden varies widely across the country. In the National Tuberculosis (TB) Prevalence survey conducted from 2019 to 2021, the estimated tuberculosis prevalence was 312 per 100,000 for all ages in India overall.^1^ The National Capital Territory of Delhi (“Delhi”) was estimated to have the highest regional tuberculosis prevalence of 747 per 100,000, whereas Gujarat was estimated to have the lowest regional tuberculosis prevalence [137 per 100,000].^1^

Tuberculosis elimination is a key focus for the Indian government, and prevention strategies, including tuberculosis vaccines and preventive treatment, are considered within the National Strategic Plan for Elimination of Tuberculosis 2017–2025.^2^ As of July 2023, there were sixteen tuberculosis vaccine candidates in clinical trials. Results are eagerly anticipated from an upcoming Phase III trial of the vaccine candidate M72/AS01_E_ and the ongoing confirmatory Phase IIb trial for BCG-revaccination, as both products have demonstrated promising results in previous Phase IIb trials.^3^^,^^4^ The Phase IIb trial of M72/AS01_E_ demonstrated 49.7% (95% confidence interval: 2.1–74.2) efficacy for preventing disease in currently infected adults, and the Phase IIb trial including BCG-revaccination estimated 45.4% (6.4–68.1) efficacy for preventing sustained *Mtb* infection in uninfected adolescents.

Earlier modelling studies have found that the introduction of new tuberculosis vaccines aligning with characteristics described in the World Health Organization (WHO) Preferred Product Characteristics for New Tuberculosis Vaccines or efficacy estimates from Phase IIb trials could have a positive impact worldwide^5^, ^6^, ^7^, ^8^, ^9^, ^10^ and in India.^11^, ^12^, ^13^, ^14^, ^15^ However, it is unknown how or if the estimated impact of tuberculosis vaccines will vary regionally within India, given the varying burdens of disease. The Indian government is set to undertake a study to investigate the impact of delivering BCG-revaccination to priority populations in 23 states.^16^ Variation in disease and infection prevalence may influence the impact of these interventions by region.

Assuming vaccine efficacy estimates from the Phase IIb trials, we used mathematical modelling to simulate hypothetical scenarios to investigate how the health impact and cost-effectiveness of M72/AS01_E_ and BCG-revaccination could vary between high- and low-tuberculosis burden areas of India—represented by Delhi and Gujarat—under varying delivery strategies and vaccine characteristics.

## Methods

### Data

Data to inform calibration was obtained from the National TB Prevalence survey in India,^1^ the India TB Report 2022 and 2023,^2^^,^^17^ and Ni-kshay—an online tuberculosis reporting and surveillance system developed by the National TB Elimination Programme.^18^ We combined available demographic data and extrapolated to obtain single age and year projections of population size for each region (Supplementary Material Section 4.3).^19^

### Model structure and calibration

We adapted a tuberculosis natural history model structure and parameterisation from previous studies.^5^^,^^11^ We employed history matching with emulation using the ‘hmer’ R package to calibrate the model to each region.^20^ We fit each model to three targets to represent the higher tuberculosis burden in Delhi, and the lower tuberculosis burden in Gujarat. We assumed a uniform distribution between lower and upper bounds, and adjusted each target as described in the Supplementary Material Sections 2 and 3. We fit to the 2021 disease prevalence per 100,000 [Delhi: 747 (510–984), Gujarat: 137 (76–198)],^1^ the 2021 notification rate per 100,000 [Delhi: 536 (429–644), Gujarat: 137 (110–165)],^17^ and the 2020 proportion of active tuberculosis that was subclinical [0.564 (interquartile range = 0.428–0.685)].^21^ The model for Gujarat was also fit to the estimated adult tuberculosis prevalence in 2011 [383 (315–451) per 100,000].^22^

### Scenarios

#### No-new-vaccine baselines

We used the calibrated models for Delhi and Gujarat to project baseline epidemiology to 2050 in each setting, assuming the coverage and quality of non-vaccine tuberculosis services continued at 2019 levels, with no new vaccine introduction, referred to as the *Status Quo* no-new-vaccine baseline.

Aligning with Clark et al.,^11^ we simulated an alternative baseline (the *Strengthened Current Interventions* no-new-vaccine baseline) for each region which assumed strengthening of current non-vaccine tuberculosis interventions between 2021 and 2035 to meet the target of a 50% reduction in tuberculosis incidence in 2035 compared to 2015.

#### Vaccine scenarios–Basecase

We simulated hypothetical *Basecase* vaccine scenarios for each vaccine product, and subsequent hypothetical alternative scenarios to investigate the impact of uncertainty in vaccine and delivery characteristics (below). *Basecase* vaccine scenario characteristics were informed by expert opinion, and by Phase IIb trial and anticipated licensure characteristics, and we assumed that the vaccine in each scenario would be delivered to an age group aligned with the clinical-trial-eligible ages.^3^^,^^4^ We did not explicitly model neonatal BCG vaccination, but assumed that it would continue to be delivered at high levels in both regions (97% coverage in Delhi and 95% coverage in Gujarat based on DHFS-5).^23^

Aligning with the mean estimate of vaccine efficacy from the Phase IIb trial, the *Basecase* M72/AS01_E_ scenario assumed a 50% efficacy prevention of disease vaccine effective with any infection status at vaccination and ten years average protection, introduced in 2030 routinely to those aged 15 (achieving 80% coverage linearly over five years) and as a campaign for ages 16–34 (achieving 70% coverage linearly over five years) in 2030 and 2040.

Aligning with the mean estimate of vaccine efficacy from the Phase IIb trial, the *Basecase* BCG-revaccination scenario assumed a 45% efficacy prevention of infection vaccine effective in individuals with no current infection (those who were uninfected with *Mtb*) at the time of vaccination and ten years average protection, introduced in 2025 routinely to those aged 10 (achieving 80% coverage linearly over five years) and as a campaign for ages 11–18 (achieving 80% coverage linearly over five years), in 2025, 2035, and 2045.

#### Vaccine scenarios–Policy Scenarios

We evaluated age-targeting *Policy Scenarios* for both vaccine products. We met with in-country partners in the Government of India to discuss preferred ages to target for tuberculosis vaccine delivery for each vaccine separately. We ensured that our hypothetical modelled scenarios captured this information to provide the most useful estimates to decision makers. The *Older Ages: M72/AS01*_*E*_ scenario assumed routine delivery to those aged 17 and a campaign for ages 18–55, and the *Older Ages: BCG-revaccination* scenario assumed routine delivery to those aged 15 and a campaign for ages 16–34. For both vaccine products, we evaluated an *All-Adults* scenario with routine delivery for those aged 18 and a campaign for everyone aged 19 and older.

#### Vaccine scenarios–Vaccine Characteristic and Coverage Scenarios

To investigate uncertainty in vaccine product characteristics, such as vaccine efficacy and mechanism of effect, we evaluated *Vaccine Characteristic and Coverage Scenarios* by varying individual characteristics of the vaccine profile from the *Basecase* (Table 1). We modelled scenarios with higher vaccine efficacies than the mean estimates used in the *Basecase* scenarios based on the upper bound of the Phase IIb trial estimates and expert opinion.

**Table 1:** Vaccine scenarios.

Characteristic	M72/AS01_E_		BCG-revaccination	
	Basecase	Univariate scenario analyses	Basecase	Univariate scenario analyses
*PolicyScenarios*				
Age targeting	Routine for age 15, campaign for ages 16–34	*Older Ages* (routine for age 17, campaign for ages 18–55) *All-Adults* (routine for age 18, campaign for ages 19+)	Routine for age 10, campaign for ages 11–18	*Older Ages* (routine for age 15, campaign for ages 16–34) *All-Adults* (routine for age 18, campaign for ages 19+)
*VaccineCharacteristic andCoverageScenarios*				
Efficacy	50%	60%, 70%	45%	70%
Mechanism of effect	Prevents disease	Prevents infection and disease	Prevents infection	Prevents infection and disease
Infection status at time of vaccination required for efficacy	Any infection (current/no current infection)	Current infection only	No current infection only	Any infection (current/no current infection)
Duration of protection	10 years	5, 15, 20	10 years	5, 15, 20
Introduction year	2030	2036	2025	2031
Achieved coverage	Medium: 80% routine, 70% campaign	Low: 70% age 15, 50% campaign High: 90% age 15, 90% campaign	Medium: 80% routine, 80% campaign	Low: 70% routine, 70% campaign High: 90% routine, 90% campaign

### Costs

We assumed vaccine delivery costs of $2.50 (1.00–5.00) per dose,^24^ supply chain costs of $0.11 (0.06–0.22) per dose,^25^ and a vaccine price of $2.50 per dose for M72/AS01_E_ (assuming two doses per course) and $0.17 per dose for BCG-revaccination (assuming one dose per course).^26^ For vaccine campaigns, we included a one-time vaccine introduction cost of $2.40 (1.20–4.80) per individual in the targeted age group to represent non-recurring start-up costs.^24^ We assumed variation in cost of vaccination time between Delhi and Gujarat due to differences in urban and rural access to healthcare (Supplementary Material Section 6.3).^25^, ^26^, ^27^, ^28^, ^29^

### Outcomes

We estimated the cumulative number of tuberculosis cases and deaths that could be averted between vaccine introduction and 2050 for each scenario compared to the predicted numbers in the no-new-vaccine baseline. We estimated potential incidence and mortality rate reductions in 2050 for each scenario compared to the estimated rates in 2050 for the no-new-vaccine baseline. We calculated incremental vaccination, diagnostic, and treatment costs for each scenario compared to the no-new-vaccine baseline in 2020 US dollars from the health-system and societal perspectives.

We performed cost-effectiveness analysis comparing the *Policy Scenarios* for each vaccine product and region. Costs and benefits were discounted to 2025 at 3% per year as per guidelines.^30^ We estimated incremental costs and disability-adjusted life years (DALYs) averted for each scenario between 2025 and 2050, using the disability weight for tuberculosis from the Global Burden of Disease 2019 study,^31^ and India-specific life expectancy estimates from the United Nations Development Programme.^32^ We calculated incremental cost-effectiveness ratios (ICERs) as mean incremental costs divided by mean incremental DALYs averted for each scenario. We evaluated the resulting ICERs against three cost-effectiveness thresholds: 1 times gross domestic product (GDP) per capita for India (US$1928), and two opportunity cost thresholds defined by Ochalek et al.: the country-level upper (US$443) and lower (US$328) bounds.^33^

To investigate if the decision to introduce a vaccine would change based on the assumed vaccine characteristics, we calculated ICERs for the *Vaccine Characteristic and Coverage Scenarios* compared to the no-new-vaccine baseline. We assumed each vaccine product was delivered using the *Basecase* age-targeting assumptions.

### Role of the funding source

The funder was involved in the development of the research question and study design, but had no role in the collection, analysis, and interpretation of the data, the writing of the report or the decision to submit the manuscript for publication.

## Results

Calibrated no-new-vaccine baseline trends for Delhi and Gujarat are in Section 8 of the Supplementary Material. Between 2025 and 2050, the *Status Quo* no-new-vaccine baseline predicted 4.1 m (95% uncertainty interval: 3.7–4.4) cases and 533 (349–761) thousand deaths in Delhi, and 2.2 m (2.0–2.5) cases and 210 (100–325) thousand deaths in Gujarat. The *Strengthened Current Interventions* no-new-vaccine baseline predicted 2.2 m (2.0–2.4) cases and 292 (192–456) thousand deaths in Delhi, and 1.7 m (1.6–1.9) cases and 179 (84–291) thousand deaths in Gujarat between 2025 and 2050. Consistent with findings from the National TB Prevalence Survey, a higher burden of disease was predicted in Delhi than in Gujarat. A lower and declining trend in tuberculosis infection prevalence was predicted in Gujarat compared to Delhi.

**Fig. 1:**
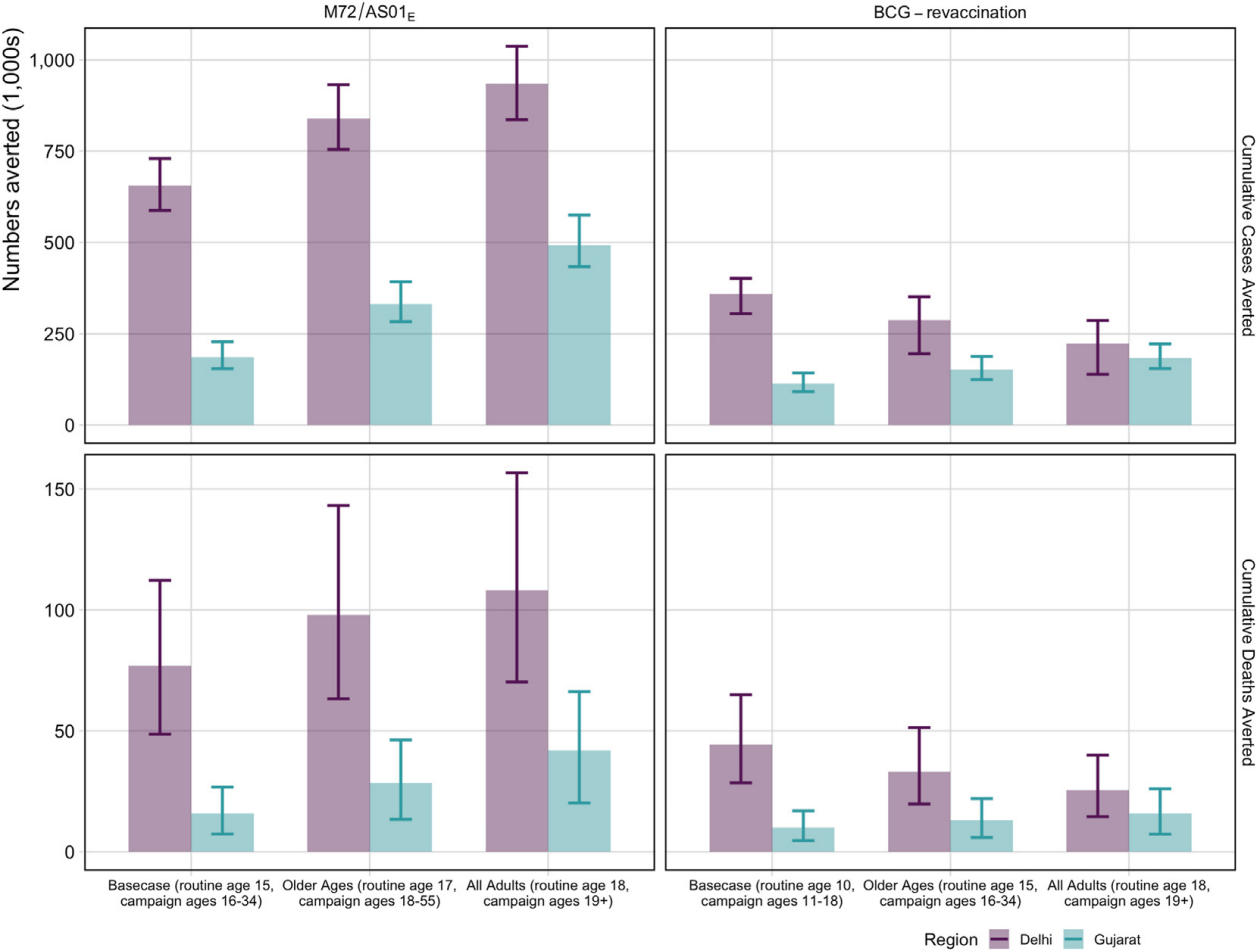
Cumulative cases and deaths averted between 2025 and 2050 for *Policy Scenarios* for both vaccines and regions. The top of each bar represents the median cumulative number of cases or deaths averted for each scenario, and the bounds represent 95% uncertainty intervals. Cumulative cases and deaths averted were compared to the predicted number of cases and deaths that would occur between 2025 and 2050 with the *Status Quo* no-new-vaccine baseline: 4.1 (3.7–4.4) million cases and 533 (349-761) thousand deaths in Delhi, and 2.2 (2.0–2.5) million cases and 210 (100–325) thousand deaths in Gujarat.

Key results estimated by the model simulations for the *Status Quo* no-new-vaccine baseline are described below, with full results in Supplementary Material Sections 8 and 9. The *Basecase* M72/AS01_E_ scenario averted 655 (587–730) thousand cases, or 16.0% of the total predicted cases, and 77 (49–112) thousand deaths, or 14.4% of the total predicted deaths between 2025 and 2050 in Delhi (Table 2). The *Basecase* M72/AS01_E_ scenario averted 186 (155–228) thousand cases (8.5% of the total predicted cases) and 16 (7–27) thousand deaths (7.6% of the total predicted deaths) in Gujarat between 2025 and 2050 (Table 2). The number of cases and deaths averted was increased in both Delhi and Gujarat with delivery to an older population (Table 2). The *All-Adults* scenario averted more cases and deaths than the *Older Ages* scenario, which similarly averted more than the *Basecase* M72/AS01_E_ scenario (Fig. 1).

**Table 2:** Health impact results for M72/AS01_E_ and BCG-revaccination in Delhi and Gujarat.

Scenario	Cumulative cases averted between 2025 and 2050 (1000s)		Cumulative deaths averted between 2025 and 2050 (1000s)		Incidence rate reduction in 2050 (%)		Mortality rate reduction in 2050 (%)	
	Delhi	Gujarat	Delhi	Gujarat	Delhi	Gujarat	Delhi	Gujarat
**M72/AS01_E_ scenarios**								
Basecase (routine age 15, campaign ages 16-34)	655 (587–730)	186 (155–228)	77 (49–112)	16 (7–27)	26 (23–29)	16 (15–19)	27 (23–30)	17 (15–19)
*PolicyScenarios*								
Older Ages (routine age 17, campaign ages 18–55)	839 (755–932)	331 (284–393)	98 (63–143)	28 (13–46)	29 (25–33)	25 (23–27)	31 (26–34)	26 (24–28)
All-Adults (routine age 18, campaign ages 19+)	935 (836–1037)	492 (434–575)	108 (70–157)	42 (20–66)	31 (26–34)	32 (30–34)	32 (27–36)	34 (32–36)
*VaccineCharacteristic andCoverageScenarios*								
Efficacy with current infection at vaccination	471 (403–535)	101 (84–124)	55 (34–82)	9 (4–15)	17 (16–19)	8 (7–9)	18 (16–20)	8 (7–9)
Prevention of infection and disease	817 (730–914)	238 (198–293)	95 (61–140)	20 (9–34)	33 (29–37)	22 (19–25)	34 (29–38)	22 (20–25)
**BCG-revaccination scenarios**								
Basecase (routine age 10, campaign ages 11-18)	359 (305–402)	113 (92–143)	44 (29–65)	10 (5–17)	13 (10–16)	10 (9–12)	14 (10–16)	10 (9–12)
*PolicyScenarios*								
Older Ages (routine age 15, campaign ages 16–34)	287 (196–352)	152 (125–188)	33 (20–51)	13 (6–22)	10 (6–14)	13 (11–15)	10 (6–14)	9 (8–11)
All-Adults (routine age 18, campaign ages 19+)	224 (139–287)	184 (155–222)	25 (15–40)	16 (7–26)	8 (4–11)	15 (13–17)	8 (4–11)	11 (10–13)
*VaccineCharacteristic andCoverageScenarios*								
Efficacy with any infection at vaccination	434 (390–494)	114 (92–145)	54 (34–80)	10 (5–17)	16 (13–19)	10 (9–12)	17 (14–19)	10 (9–12)
Prevention of infection and disease	544 (490–601)	154 (125–195)	67 (43–98)	14 (6–23)	21 (17–24)	14 (12–16)	21 (18–24)	13 (12–16)

Estimates are provided as the median and 95% uncertainty intervals.

If M72/AS01_E_ was able to prevent both infection and disease, the number of cases and deaths averted could increase by 23–25% in Delhi and 25–28% in Gujarat compared to the *Basecase* M72/AS01_E_ scenario (Table 2). However, if M72/AS01_E_ was only efficacious in individuals with *Mtb* infection at the time of vaccination (“current infection”), the number of cases and deaths averted could decrease by 28–29% in Delhi and 44–46% in Gujarat compared to the *Basecase* M72/AS01_E_ scenario (Table 2).

The *Basecase* BCG-revaccination scenario averted 359 (305–402) thousand cases (8.8% of total predicted cases) and 44 (29–65) thousand deaths (8.3% of total predicted deaths) in Delhi, and 113 (91–143) thousand cases (5.1% of total predicted cases) and 10 (5–17) thousand deaths (4.8% of total predicted deaths) in Gujarat between 2025 and 2050 (Table 2). Due to differences in modelled infection prevalence, delivering BCG-revaccination to an older population (*Older Ages* and *All-Adults* scenarios) decreased the number of cases and deaths averted in Delhi, but increased the impact in Gujarat compared to the *Basecase* BCG-revaccination scenario (Fig. 1).

If BCG-revaccination was able to prevent infection and disease, the absolute number of cases and deaths averted could increase by 52–53% in Delhi and 36–40% in Gujarat compared to the *Basecase* BCG-revaccination scenario (Table 2). If BCG-revaccination worked in any infection status opposed to only those who were uninfected, the number of cases and deaths averted could increase by 21–23% in Delhi, but could only increase the number of cases and deaths averted in Gujarat by 0–1% compared to the *Basecase* BCG-revaccination scenario (Table 2).

In both regions, M72/AS01_E_ resulted in a higher number of cases and deaths averted than BCG-revaccination: approximately 1.8 times in Delhi and 1.6 times in Gujarat (Table 2). For both vaccine products, more cases and deaths were averted in Delhi compared to Gujarat: 3.5–4.8 times for M72/AS01_E_ and 3.2–4.4 times for BCG-revaccination (Table 2).

The total vaccination cost for the M72/AS01_E_ *Basecase* was US$118 m (80–173) in Delhi and was US$366 m (248–536) in Gujarat, compared to the BCG-revaccination *Basecase* total vaccination cost of US$27 m (12–49) in Delhi and US$97 m (42–178) in Gujarat (Tables S10.2, S10.5, S10.8, S10.11). Larger vaccination costs were predicted for introducing M72/AS01_E_ compared to BCG-revaccination in both regions: 4.4 times more in Delhi and 3.8 times more in Gujarat. Incorporating cost-savings in treatment and diagnostic costs, the total incremental programme cost for the M72/AS01_E_ *Basecase* in Delhi was US$5 m (minus 37–63) and in Gujarat was US$332 m (213–505) (Tables S10.2, S10.8). The *Basecase* BCG-revaccination scenario led to cost-savings of US$38 m (58–13) in Delhi (Table S10.5). The total programme cost for the *Basecase* BCG-revaccination scenario in Gujarat was US$77 m (21–158) in Gujarat (Table S10.11).

**Fig. 2:**
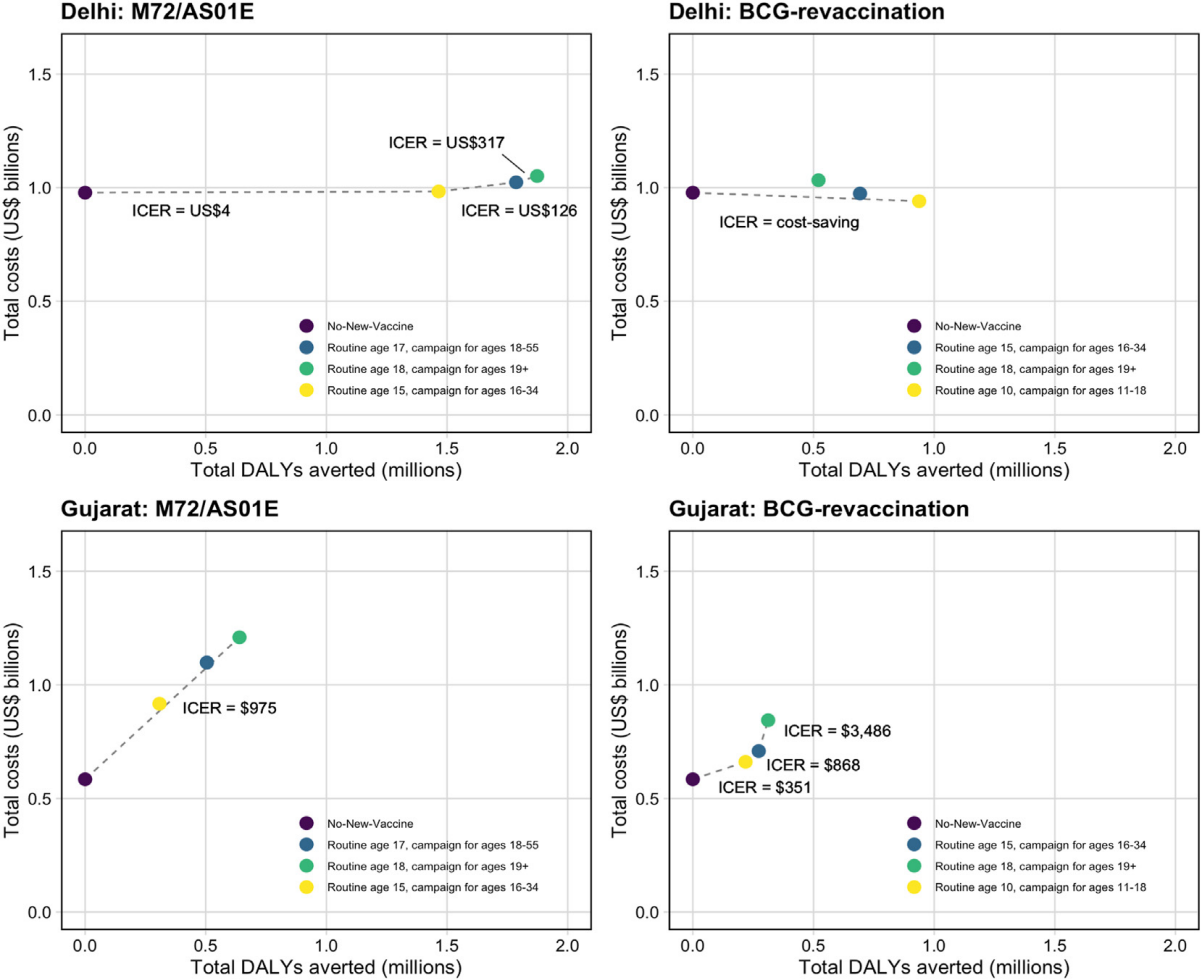
Competing choice cost-effectiveness analysis for Delhi and Gujarat *Policy Scenarios* for both vaccine products.

In Delhi, introducing M72/AS01_E_ was potentially cost-effective for all *Policy Scenarios*. The *Basecase* M72/AS01_E_ scenario (ICER = US$4), *Older Ages* scenario (ICER = US$126) and *All-Adults* scenario (ICER = US$317) were cost-effective at the country-level upper and lower bounds, and the 1 times GDP threshold (Table 3, Fig. 2). The incremental cost of the *Basecase* M72/AS01_E_ scenario was US$5 m (minus 37–63), averting 1.5 m (1.0–2.1) DALYs between 2025 and 2050 compared to the no-new-vaccine baseline (Table 3, Fig. 2). In Gujarat, only the *All-Adults* scenario was considered potentially cost-effective for M72/AS01_E_ at the 1 times GDP threshold (ICER = US$975) (Table 3, Fig. 2). The cost of the *All-Adults* scenario compared to the no-new-vaccine baseline was US$624 m and 640 thousand DALYs were averted between 2025 and 2050 (Table 3, Fig. 2).

**Table 3:** Competing choice cost-effectiveness analysis for Delhi and Gujarat.

Scenario	Total costs (USD, 1000s)	Total DALYs averted (1000s)	Incremental cost (USD, 1000s)	Incremental DALYs averted (1000s)	Cost (USD) per DALY averted
**Delhi**					
***M72/AS01***_***E***_***PolicyScenarios***					
No-new-vaccine	977,788	–	–	–	–
Basecase (routine age 15, campaign for ages 16–34)	982,966	1465	5178	1465	4
Older Ages (routine age 17, campaign for ages 18–55)	1,023,279	1786	40,313	321	126
All-Adults (routine age 18, campaign for ages 19+)	1,050,875	1873	27,596	87	317
***BCG-revaccination******P******olicyScenarios***					
No-new-vaccine	977,788	–	–	–	–
Basecase (routine age 10, campaign for ages 11–18)	940,220	938	−37,568	938	*Cost-saving*
Older Ages (routine age 15, campaign for ages 16–34)	973,930	693	–	–	*Strongly dominated*
All-Adults (routine age 18, campaign for ages 19+)	1,032,616	521	–	–	*Strongly dominated*
**Gujarat**					
***M72/AS01***_***E***_***P******olicyScenarios***					
No-new-vaccine	584,609	–	–	–	–
Basecase (routine age 15, campaign for ages 16–34)	917,077	308	–	–	*Weakly dominated*
Older Ages (routine age 17 campaign for ages 18–55)	1,097,770	505	–	–	*Weakly dominated*
All-Adults (routine age 18, campaign for ages 19+)	1,208,573	640	623,965	640	975
***BCG-revaccinationPolicyScenarios***					
No-New-Vaccine	584,609	–	–	–	–
Basecase (routine age 10, campaign for ages 11–18)	661,265	219	76,656	219	351
Older Ages (routine age 15, campaign ages 16–34)	708,672	273	47,407	55	868
All-Adults (routine age 18, campaign ages 19+)	844,338	312	135,666	39	3486

In Delhi, the *Older Ages* and *All-Adults* BCG-revaccination scenarios were dominated by the *Basecase* BCG-revaccination scenario. The *Basecase* BCG-revaccination scenario was considered cost-effective at all thresholds (ICER = cost-saving), with cost-savings of US$37 m and averted 938 thousand DALYs between 2025 and 2050 compared to the no-new-vaccine baseline. In Gujarat, the *Basecase* BCG-revaccination scenario was cost-effective at the country-level upper bound (ICER = US$351), with an incremental cost of US$77 m compared to the no-new-vaccine baseline and averted 219 thousand DALYs between 2025 and 2050. The *Older Ages* scenario was cost-effective at 1 times GDP per capita (ICER = US$868) (Table 3, Fig. 2).

**Fig. 3:**
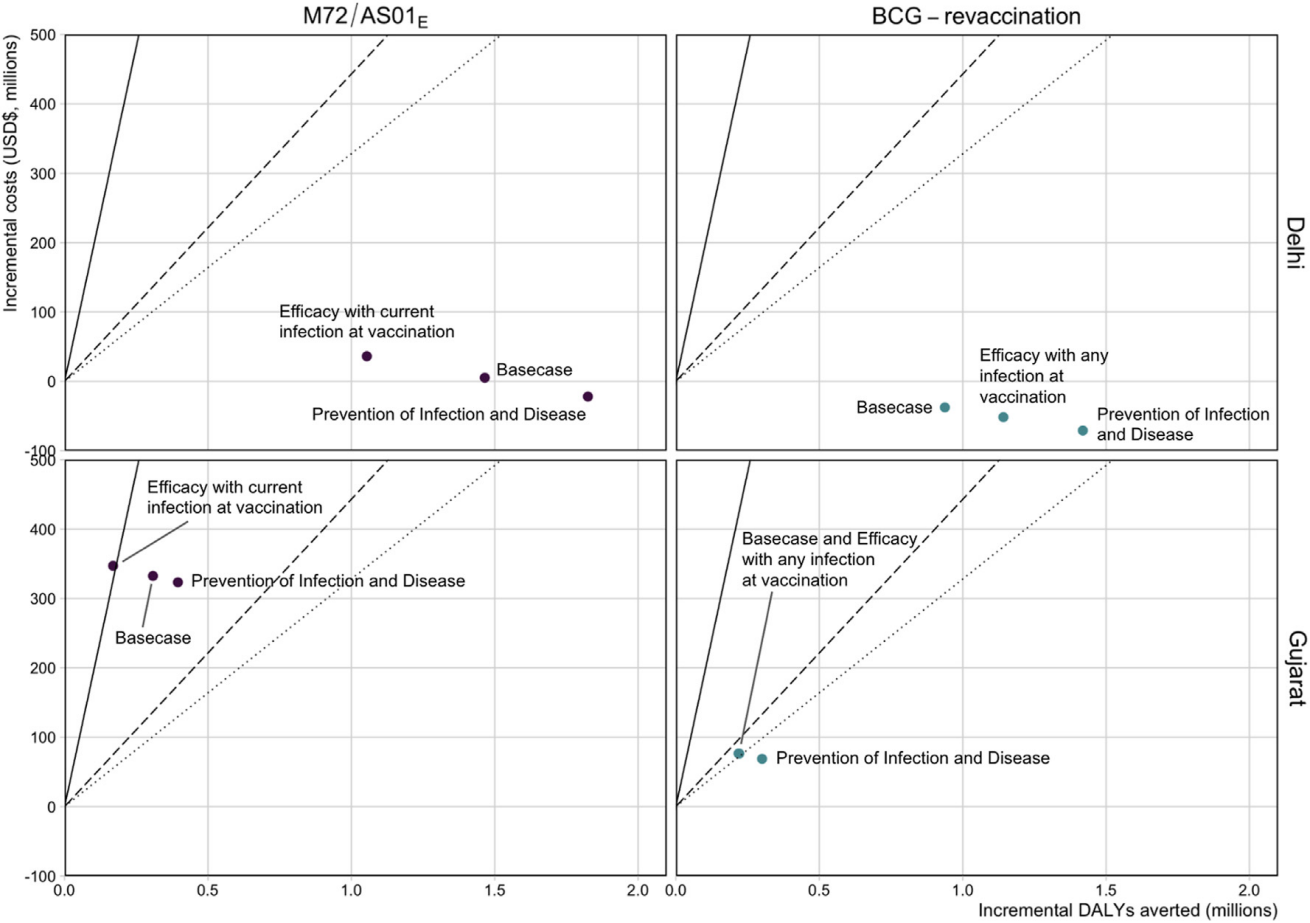
Comparison of ICERs for select *Vaccine Characteristic and Coverage Scenarios*. The cost-effectiveness thresholds are indicated as follows: solid line = 1 times GDP per capita (US$1928), dashed line = country-level upper bound (US$443), and dotted line = country-level lower bound (US$328). The *Basecase* M72/AS01_E_ scenario assumes a 50% efficacy POD vaccine efficacious with any infection status at the time of vaccination, with 10 years duration of protection reaching 80% coverage for 15-year-olds and 70% coverage for those aged 16–34. Each M72/AS01_E_ scenario is delivered routinely to those aged 15 and as a campaign for those aged 16–34. The *Basecase* BCG-revaccination scenario assumes a 45% efficacy POI vaccine efficacious with no current infection at the time of vaccination, with 10 years duration of protection and reaching 80% coverage. Each BCG-revaccination scenario is delivered routinely to those aged 10 and as a campaign for those aged 11–18. The scenarios on the figure are labelled with the difference in product characteristics for that scenario compared to the *Basecase*.

When comparing the ICERs from the *Vaccine Characteristic and Coverage Scenarios* in Delhi, regardless of the assumed product characteristics, introducing M72/AS01_E_ routinely to those aged 15 and as a campaign for ages 16–34 could be cost-effective, and in some cases, cost-saving, at the country-level lower bound (Fig. 3). Similarly, introducing BCG-revaccination routinely to those aged 10 and as a campaign for ages 11–18 could be cost-saving in Delhi (Fig. 3). In Gujarat, delivering M72/AS01_E_ routinely to those aged 15 and as a campaign for ages 16–34 could be cost-effective at a 1 times GDP per capita threshold, except if the vaccine was only efficacious with current infection at vaccination (Fig. 3). Introducing BCG-revaccination in Gujarat could be cost-effective regardless of the assumed product characteristics (Fig. 3).

In both regions, there were larger ICERs for M72/AS01_E_ scenarios compared to BCG-revaccination, and for both vaccine products, larger ICERs for Gujarat compared to Delhi (Fig. 3). The total number of vaccine doses delivered for each region and scenario is included in the Supplementary Material Section 10.

With the *Strengthened Current Interventions* no-new-vaccine baseline, the *Basecase* M72/AS01_E_ scenario could avert 229 thousand (151–305) cases and 25 thousand (14–45) deaths in Delhi, and 111 thousand (68–167) cases and 10 thousand (4–21) deaths in Gujarat between 2025 and 2050, corresponding to averting 6.1–12.8% of cases and deaths in Delhi and 3.7–9.0% of cases and deaths in Gujarat during the time period. The *Basecase* BCG-revaccination scenario could avert 115 thousand (55–171) cases and 14 thousand (6–25) deaths in Delhi, and 63 thousand (30–113) cases and 6 thousand (2–15) deaths in Gujarat between 2025 and 2050, corresponding to averting 2.4–7.3% of cases and deaths in Delhi and 1.8–6.0% of cases and deaths in Gujarat during the time period.

Full impact results are in Supplementary Material Sections 9 and 10.

## Discussion

Our modelling suggests that, assuming vaccine efficacy aligned with the mean estimates from the Phase IIb trials, hypothetical scenarios of introducing M72/AS01_E_ and BCG-revaccination could have a positive impact in Delhi and Gujarat given the assumed vaccine and delivery characteristics. M72/AS01_E_ scenarios resulted in a higher number of cases and deaths averted than BCG-revaccination in both regions, and more cases and deaths were averted in Delhi compared to Gujarat. We found that given the assumed characteristics, both products were likely to be cost-effective or cost-saving in Delhi. In Gujarat, M72/AS01_E_ was likely to be cost-effective unless it only worked in those with current infection at the time of vaccination. M72/AS01_E_ scenarios had higher vaccination costs than BCG-revaccination, and higher vaccination costs were estimated in Gujarat overall than in Delhi.

For all modelled hypothetical scenarios, M72/AS01_E_ could have a larger and faster impact on the tuberculosis burden (more cases and deaths averted compared to the no-new-vaccine baseline) than BCG-revaccination. We assumed that M72/AS01_E_ would be effective regardless of the presence or absence of infection, and work by preventing disease. Therefore, those with current infection who received the vaccine would immediately have a lower rate of disease progression. We assumed that BCG-revaccination would only be effective in those who were uninfected at vaccination and would work by preventing infection. Therefore, the impact from BCG-revaccination on cases and deaths averted would be delayed by the typical time from vaccination to infection, and the typical time from infection to disease.

Several findings related to the lower infection prevalence modelled in Gujarat compared to Delhi. For M72/AS01_E_ scenarios, the relative decrease in the number of cases and deaths averted if M72/AS01_E_ was only effective in individuals with current infection was much larger in Gujarat compared to Delhi. If M72/AS01_E_ vaccine efficacy was restricted to those with current infection, a larger proportion of the population would no longer benefit from vaccination in Gujarat compared to Delhi, due to the lower infection prevalence in Gujarat. BCG-revaccination was estimated to have a larger relative impact in Gujarat than in Delhi for strategies targeting an older and larger proportion of the population (*Older Ages* or *All-Adults* scenarios compared to the *Basecase*). As we modelled a higher infection prevalence for all ages in Delhi, and assumed that BCG-revaccination would only be effective if administered to people who were uninfected, there was a higher proportion of the population who were uninfected and would receive protection from the vaccine in Gujarat than in Delhi.

Across the range of assumptions examined for vaccine product characteristics, M72/AS01_E_ and BCG-revaccination were likely to be cost-effective (and even cost-saving) in Delhi compared to the thresholds evaluated. In Gujarat, M72/AS01_E_ could be cost-effective unless efficacy was restricted to those with current infection, and BCG-revaccination was likely to be cost-effective regardless of the modelled characteristics. National-level modelling of M72/AS01_E_ and BCG-revaccination in India demonstrated similar results, with M72/AS01_E_ likely to be cost-effective at the lowest cost-threshold except for when efficacy was restricted to those with current infection, and BCG-revaccination likely to be highly cost-effective across all scenarios evaluated. Understanding the mechanism of effect of M72/AS01_E_ and confirming whether it works in all populations is a key area for future research, particularly in Gujarat and other areas with a low prevalence of infection.

M72/AS01_E_ was predicted to have higher vaccination costs than BCG-revaccination in both regions: 4.4 times as high in Delhi (US$118 m vs US$27 m) and 3.5 times as high in Gujarat (US$366 m vs US$97 m), due to the higher price per dose for M72/AS01_E_, ($2.50 per dose vs $0.17 per dose for BCG) and assuming two doses per course. Higher costs for both products were predicted in Gujarat compared to Delhi due to the larger population size.

There are limitations associated with this work. Firstly, this is a hypothetical mathematical modelling study, and therefore limitations associated with models apply. We represented tuberculosis natural history with a compartmental model accounting for multiple infection states. If our assumptions around how the latency structure or aspects such as subclinical tuberculosis interact with vaccines were incorrect, we may have over- or under-protected the population, leading to incorrect impact estimates. We assumed bounds of certain natural history parameters would not vary between regions in India, and therefore used national India posterior ranges as prior ranges for Delhi and Gujarat calibration.^11^ As the true distributions remain unknown, we assumed a uniform distribution between upper and lower bounds for the natural history parameter prior ranges, to allow the calibration procedure (history matching with emulation) to capture all aspects of model behaviour across the relevant parameter space, ensuring robust calibration. If this was an incorrect assumption, or if initial assumptions on the national India model prior ranges were incorrect, our projections may inaccurately represent Delhi and Gujarat.

Our model included an on-treatment compartment but assumed the only people treated were those with disease. The reported notification rate in Gujarat was greater than the prevalence estimate, implying more people were treated per year than those with prevalent disease. While Gujarat has excellent tuberculosis treatment services, only 35% of reported notifications in 2021 were bacteriologically confirmed. Therefore, there could be treatment of individuals who did not have tuberculosis, which we did not represent, but could be investigated with future adaptations to the model.

A key limitation of this work was the availability of region-specific data to inform calibration. The National TB Prevalence Survey in India provided estimates of the tuberculosis prevalence for each region for one year, allowing us to model a higher burden of tuberculosis in Delhi compared to Gujarat, but this did not allow us to incorporate a data-driven time trend. There were no region-specific calibration targets to constrain mortality, and therefore we found large uncertainty on the number of cumulative deaths averted due to large uncertainty around trends in mortality. Additionally, there were no region-specific estimates of infection prevalence, which was a key determiner of vaccine impact. We assumed that differences in mortality and infection prevalence between Delhi and Gujarat would align with the differences observed in disease prevalence and modelled a higher mortality rate and infection prevalence in Delhi. There was limited data available for subnational regions to dynamically inform service disruptions caused by the COVID-19 pandemic, and therefore it is possible that the actual future trends observed in Delhi and Gujarat will not align with our simulations. As subnational estimates of future burden become available (e.g., from WHO or the Institute for Health Metrics and Evaluation), these could be used as additional sources of evidence to compare with future burden predicted by baseline scenarios.

We represented population size and age structure for Delhi and Gujarat by utilising all available demographic data and projections for the regions and extrapolated forward from 2037 to 2050 where no data was available. As the risk of tuberculosis is age-dependent, if we incorrectly represented the demographic structure or population size of the regions we may have slightly over or underestimated the health impact and cost-effectiveness of new vaccines. To continue modelling subnational regions, more region-specific data to inform model predictions is urgently needed.

The *Strengthened Current Interventions* no-new-vaccine baseline assumed that there would be scale-up in the currently available tools and technologies to reduce progression to tuberculosis disease to hit 50% of the tuberculosis incidence rate in 2015 in 2035, but did not consider specific interventions for this to be done. We introduced vaccines into the population independently, and did not integrate with other available services, such as tuberculosis preventive therapy, which may alter future outcomes.

The potential introduction years used in this study were based on the real-world potential availability and feasibility of introducing BCG-revaccination and M72/AS01_E_. Given that BCG is already licensed, introduction could require a policy change to deliver to adolescents, therefore leading to an earlier introduction year. However, M72/AS01_E_ is still undergoing a Phase III trial, and therefore likely to be introduced later. If M72/AS01_E_ was introduced for 25 years (as the *Basecase* BCG-revaccination scenario), the health impact would be higher. As we already observed that the health impact by 2050 was greater for M72/AS01_E_ than BCG-revaccination, the results would continue to support our findings.

The purpose of this paper was to provide a hypothetical estimate of the value and impact of introducing M72/AS01_E_ or BCG-revaccination given the specific vaccine efficacy values and additional vaccine characteristics assumed, as opposed to directly informing policy decisions surrounding vaccine rollout. We presented hypothetical scenarios with vaccine efficacy at or above the mean estimates from the M72/AS01_E_ and BCG-revaccination Phase IIb trials and did not showcase scenarios with lower efficacy. A confirmatory Phase IIb trial for BCG-revaccination and a Phase III trial for M72/AS01_E_ are currently underway, which will provide further evidence on the vaccine efficacy of both vaccines and will narrow the range of uncertainty on the efficacy estimate. If results from the trials suggested a lower vaccine efficacy, it is unlikely that the vaccine would be introduced.

We assumed that the *Basecase* M72/AS01_E_ scenario would be effective with any infection status at the time of vaccination, aligning with the anticipated indicated population and studies which have demonstrated an immune response in those who were uninfected. However, the Phase IIb trial, which informed the 50% efficacy estimate, only enrolled individuals who were IGRA positive. Therefore, we evaluated a scenario where M72/AS01_E_ was only effective in those with current infection at the time of vaccination and determined that efficacy in those who are uninfected is an important driver of health impact and cost-effectiveness, particularly in populations with a lower infection prevalence.

Although we assumed BCG-revaccination would only be effective if delivered to those who were uninfected at the time of vaccination, we did not assume any “pre-vaccination infection testing”, and simulated delivery for everyone within the targeted age group. Therefore, a subset of vaccinated individuals would not receive protection due to their infection status, and the doses delivered would be wasted. Future analyses could estimate the cost trade-off between providing inefficient doses and performing pre-vaccination infection testing to ensure only those who would benefit from the vaccine receive it. We accounted for uncertainty in vaccine delivery and introduction costs but simulated a fixed vaccine price for M72/AS01_E_ and BCG-revaccination. As vaccine price dominates the cost structure, future studies could investigate cost-effectiveness with alternative vaccine prices. Additionally, we measured cost-effectiveness against national-level thresholds for India, which may not be appropriate to extrapolate to Delhi and Gujarat, and this could be an area of future study.

Our hypothetical study has demonstrated that M72/AS01_E_ and BCG-revaccination could be impactful and cost-effective if introduced in Delhi and Gujarat given the assumptions made on vaccine efficacy and other vaccine profile and delivery characteristics. Delhi and Gujarat were selected as the modelled regions to represent a high and low burden setting respectively, but future modelling studies for other regions in India could provide beneficial information for subnational decision-makers when considering vaccine introduction (model code is available). There were differences in the estimated vaccine impact between regions, which were only revealed through subnational modelling and considering differences in disease and infection prevalence. While national models are beneficial to demonstrate potential impact overall, if there are distinct epidemiological differences within a country the impact can vary.

Our results support the need for more infection prevalence surveys. We discovered how important the modelled infection prevalence of each region was to determine the likely impact if vaccines may only work in those who are uninfected or those who are infected. Age-specific regional estimates of infection prevalence would help to inform delivery strategies for vaccines only effective in people with a particular infection status, and improve estimates of vaccine impact. Another key area for future research is investigating the mechanism of effect of M72/AS01_E_, and confirming effectiveness in uninfected individuals, which was an important driver of impact and cost-effectiveness in Gujarat. Further research to reduce vaccine characteristic uncertainty (particularly uncertainty in vaccine efficacy estimates) and generate subnational models for additional regions is needed to maximise success of vaccine delivery in India.

## Contributors

Conception: RAC, AP, CKW, RCH, NAM, RGW.

Data acquisition and preparation: RAC, AP, CKW, RGW, NAM.

Data analysis: RAC, AP, TS, CKW, NAM, RGW.

Interpretation of results: RAC, AP, RGW, NAM, TS, KR, SKM, DT, CKW.

Manuscript drafting and revisions: RAC, AP, RGW, NAM, TS, KR, SKM, DT, CKW, RB, RCH.

All authors had the opportunity to access and verify the data and were responsible for the decision to submit the manuscript for publication.

## Data sharing statement

No individual level participant data was used for this modelling study. Analytic code will be made available at https://doi.org/10.5281/zenodo.6421372 immediately following publication indefinitely for anyone who wishes to access the data for any purpose.

## Declaration of interests

RCH reports employment by Sanofi Pasteur, unrelated to tuberculosis and outside the submitted work. NAM received consulting fees from The Global Fund to Fight AIDS, Tuberculosis and Malaria, and WHO, and reports funding to their institution from the U.S. Centers for Disease Control and Prevention, the Bill & Melinda Gates Foundation, NIH, and U.S. Council of State and Territorial Epidemiologists. RGW is also funded for other work by the Wellcome Trust (218261/Z/19/Z), NIH (1R01AI147321-01), EDCTP (RIA208D-2505 B), UK MRC (CCF 17-7779 via SET Bloomsbury), ESRC (ES/P008011/1), BMGF (OPP1084276, OPP1135288 & INV-001754), and WHO. All other authors declare no conflicts of interest.
